# Efficacy and safety of pharmacologic therapies in acromegaly: a systematic literature review and network meta-analysis

**DOI:** 10.1210/clinem/dgag085

**Published:** 2026-03-03

**Authors:** Roberto Salvatori, Raffaella M Colzani, Noemi Hummel, Agnieszka Kopiec, Zuzanna Maliszewska, Suwei Wang, Janetricks C Okeyo, Robert M Cuddihy, Lisa B Nachtigall

**Affiliations:** Division of Endocrinology, Diabetes and Metabolism, Department of Medicine, and Pituitary Center, The Johns Hopkins University School of Medicine, Baltimore, MD 21287, USA; Crinetics Pharmaceuticals, Inc., San Diego, CA 92121, USA; Certara GmbH, 79539 Lörrach, Germany; Certara, 30-720 Kraków, Poland; Certara, 30-720 Kraków, Poland; Crinetics Pharmaceuticals, Inc., San Diego, CA 92121, USA; Crinetics Pharmaceuticals, Inc., San Diego, CA 92121, USA; Crinetics Pharmaceuticals, Inc., San Diego, CA 92121, USA; Division of Endocrinology, Diabetes and Metabolism, Mayo Clinic, Rochester, MN 55905, USA; Neuroendocrine and Pituitary Tumor Clinical Center, Massachusetts General Hospital, Boston, MA 02114, USA

**Keywords:** acromegaly, paltusotine, network meta-analysis, systematic literature review, biochemical control, discontinuation rate

## Abstract

**Context:**

There are limited head-to-head trials comparing pharmacological treatments for acromegaly.

**Objective:**

Systematically review the efficacy and safety of pharmacological treatments for acromegaly and conduct a network meta-analysis (NMA) enabling indirect comparisons.

**Methods:**

MEDLINE and Embase were searched to identify randomized controlled trials (RCTs) of acromegaly therapies. Screening and data extraction followed PRISMA guidelines. Feasibility assessment evaluated homogeneity and consistency assumptions required for NMA. Bayesian NMAs estimated relative treatment effects and ranking probabilities.

**Results:**

Twenty-two records covering 18 RCTs were included. Biochemical control rates were comparable among long-acting injectable somatostatin receptor ligands (SRLs), including lanreotide autogel (LAN-ATG), octreotide long-acting release (OCT-LAR), pasireotide, the GH receptor antagonist pegvisomant, oral octreotide (O-OCT), octreotide subcutaneous depot (SC-OCT-D), and the once-daily oral SRL paltusotine. Paltusotine demonstrated significantly higher biochemical control vs O-OCT and SC-OCT-D (odds ratios [ORs], 95% credible intervals [CrIs]: 7.34 [1.48-36.07] and 7.85 [1.72, 36.25]). Pasireotide showed significantly higher biochemical control vs OCT-LAR (OR: 2.03 [1.29-3.23]). Paltusotine had significantly lower discontinuations due to adverse events (AEs) vs O-OCT and SC-OCT-D, (ORs: 0.022 [0.001-0.424] and 0.022 [0.001-0.343]), with similar rates to other treatments. Treatment-emergent AEs (TEAEs) and serious TEAEs were comparable across treatments. Rankings suggested paltusotine as the treatment with the highest probability of ranking as the most effective (or tolerable) treatment across all endpoints studied.

**Conclusion:**

This systematic review and NMA consolidate recent high-quality RCT evidence for acromegaly treatments. Paltusotine emerges as a promising alternative to injectable SRLs, with favorable efficacy, safety, and AE-related discontinuation patterns. These findings may inform clinical decision-making and guideline development, if confirmed by clinical experience.

Acromegaly is a rare, chronic endocrine disorder in which excessive secretion of growth hormone (GH), usually from a pituitary adenoma, causes increased hepatic and local production of insulin-like growth factor 1 (IGF-I) (1). Prolonged exposure to these hormones results in progressive somatic changes, metabolic dysfunction, and complications across multiple organ systems, contributing to increased morbidity and mortality. Acromegaly typically manifests in adulthood, with diagnosis often delayed by nearly a decade due to the insidious progression of symptoms and limited disease awareness (2). Epidemiological studies report a global diagnosed prevalence of approximately 5.9 per 100 000 persons and an annual incidence of 0.38 per 100 000 person-years, confirming acromegaly as a rare disease under international definitions (1, 3). The primary treatment objectives for acromegaly are to normalize GH and IGF-I levels, minimize symptoms, control tumor growth, and mitigate comorbidities (4). Transsphenoidal surgery is considered the preferred first-line option; however, some patients are not suitable candidates for surgery due to tumor characteristics or comorbidities, and many require long-term medical therapy due to incomplete surgical remission (5). Somatostatin receptor ligands (SRLs), including octreotide long-acting release (SANDOSTATIN® LAR; OCT-LAR) (6), lanreotide autogel (SOMATULINE® depot; LAN-ATG) (7), and pasireotide (SIGNIFOR® LAR) (8), are standard pharmacologic treatments that achieve biochemical control in a substantial proportion of patients (9). Additional therapeutic options include the GH receptor antagonist pegvisomant (SOMAVERT®) (10) and oral dopamine agonists such as the off-label agent cabergoline (11-13). While effective, these therapies are associated with well-documented limitations, including injection burden, gastrointestinal adverse events (AEs), metabolic complications, and inadequate biochemical control in a subset of patients (14).

Recent therapeutic advances have aimed to overcome these limitations by improving efficacy, tolerability, and treatment convenience. An oral SRL formulation, twice-daily octreotide capsules (MYCAPSSA®; O-OCT) (15), was approved for long-term maintenance therapy in patients who have responded to and tolerated injectable SRLs (16). Paltusotine (PALSONIFY^TM^) (17) an oral, once-daily, nonpeptide, selective somatostatin 2 receptor (SST2) agonist was recently approved by the US Food and Drug Administration (FDA) for the treatment of adults with acromegaly who had an inadequate response to surgery and/or for whom surgery is not an option (17, 18). In addition, CAM2029 (OCZYESA®; SC-OCT-D), a subcutaneous OCT depot formulation, is currently under US FDA review and was approved by the European Medicine Agency for maintenance treatment in patients who have responded to and tolerated treatment with somatostatin analogs (19). Three recently published phase 3 randomized controlled trials, CHIASMA OPTIMAL (O-OCT), PATHFNDR-1 (paltusotine), and ACROINNOVA 1 (SC-OCT-D), are similar in design, IGF-I measurement methodology, endpoints, and objective: evaluating alternative therapies for SRL-stabilized maintenance in patients with acromegaly (16, 20, 21). Each trial demonstrated efficacy and tolerability, supporting the potential of an oral or subcutaneous SRL to reduce treatment burden and enhance patient convenience (Fig. 1). Given the scarcity of head-to-head trials, indirect treatment comparisons are particularly valuable to contextualize emerging treatments within the broader treatment landscape.

**Figure 1:**
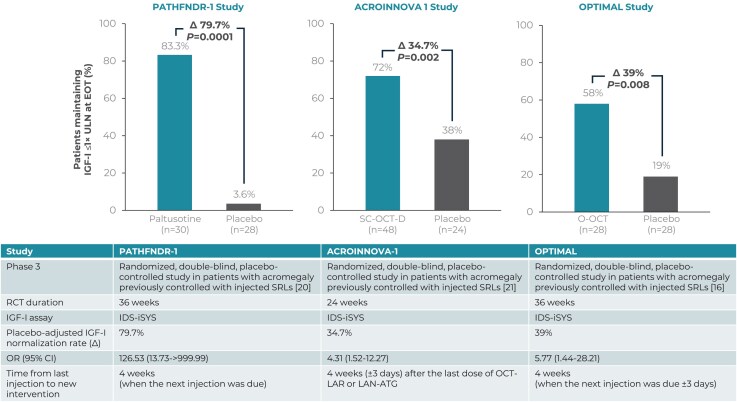
Efficacy of switching to oral paltusotine, subcutaneous octreotide depot, and oral octreotide in phase 3 acromegaly trials designed to assess maintenance of biochemical control in patients biochemically controlled on injectable SRLs. Abbreviations: ATG, autogel; CI, confidence interval; IDS-iSYS, immunoassay system for measuring IGF-I; IGF-I, insulin-like growth factor 1; LAN, lanreotide; LAR, long-acting release; OCT, octreotide; O-OCT, oral octreotide; OR, odds ratio; *P*, *P*-value; RCT, randomized controlled trial; SC-OCT-D, subcutaneous octreotide depot; SRL, somatostatin receptor ligand; ULN, upper limit of normal.

A comprehensive review of randomized clinical trials (RCTs) of acromegaly treatments was published in 2018 (22), prior to the availability of pivotal data on O-OCT, paltusotine, and SC-OCT-D. More recent systematic literature reviews (SLRs) and network meta-analyses (NMAs) completed up to June 2024 did not include the latest trials of paltusotine and SC-OCT-D or employed different methodologies, such as inclusion of observational data (23, 24). Therefore, this SLR was designed to update and expand the evidence base for pharmacological therapies for acromegaly.

## Materials and methods

### Systematic literature review

#### Literature search

This SLR was conducted in accordance with current best practices for evidence synthesis in rare endocrine disorders and is reported following the PRISMA 2020 guidelines (25). A predefined protocol specified the research objectives, eligibility criteria, search strategy, study selection process, data extraction, and risk-of-bias assessment, enhancing transparency and reproducibility.

The objectives of the SLR were 2-fold: (1) to update the evidence base established by Leonart et al (2018) (22) by identifying RCTs of pharmacological treatments for acromegaly published from 2017 through April 2025, and (2) to identify RCTs evaluating other relevant therapies not included in the Leonart review, regardless of publication date.

Eligibility criteria were defined using the population, intervention, comparator, outcomes, and study design (PICOS) (26) framework (Table 1). The patient population of interest was adults with acromegaly. Eligible interventions included cabergoline, LAN-ATG, LAN-PR/SR, OCT-LAR, O-OCT, paltusotine, pasireotide, pegvisomant, and SC-OCT-D, administered as monotherapy or in combination. Comparators included placebo or other active pharmacological treatments. Primary outcomes were the proportion of patients achieving biochemical control and safety outcomes (treatment discontinuation due to AEs, incidence of any treatment-emergent AEs (TEAEs), or serious TEAEs). Only RCTs were included, encompassing phase 2 studies with a comparator and all phase 3 or later trials. Eligible publication types included peer-reviewed full-text manuscripts and conference abstracts or proceedings in English. Observational, nonrandomized, preclinical, or narrative studies were excluded.

**Table 1:** PICOS eligibility criteria

Component	Description
**Population**	Adults with acromegaly
**Intervention**	Cabergoline, LAN-ATG, LAN-PR/SR, OCT-LAR, O-OCT, pasireotide LAR, pegvisomant, paltusotine, SC-OCT-D (monotherapy or combination)
**Comparator**	Placebo or other active pharmacological treatments
**Outcomes**	**Efficacy:** IGF-I ≤ 1.0× ULN, GH <2.5 μg/L + normal IGF-I, change in IGF-I (ULN), IGF-I < 1.3× ULN, GH <1.0 ng/mL
	**Safety:** Discontinuation, any AE/SAE, diabetes/hyperglycemia, diarrhea, abdominal pain, cholelithiasis, headache, fatigue
**Study Design**	RCTs (phase 2 with comparator, phase 3 or later)
**Language**	English

Abbreviations: AE, Adverse Event; GH, Growth Hormone; IGF-I, Insulin-like Growth Factor 1; LAN-ATG, lanreotide autogel; LAN-PR/SR, lanreotide prolonged/sustained release; LAR, Long-Acting Release; NMA, Network Meta-Analysis; OCT-LAR, octreotide long-acting release; O-OCT, oral octreotide capsules; PICOS, Population, Intervention, Comparator, Outcomes, Study Design; RCT, Randomized Controlled Trial; SAE, Serious Adverse Event; SC-OCT-D, subcutaneous octreotide depot; SD, Standard Deviation; ULN, Upper Limit of Normal.

Systematic searches were conducted in MEDLINE and Embase via Ovid on April 17, 2025, combining controlled vocabulary (MeSH/Emtree) and free-text terms for acromegaly, interventions, and RCT design. No geographical restrictions were applied. Reference lists of relevant reviews were screened to identify additional eligible trials.

#### Screening

Screening occurred in 2 stages. First, titles and abstracts were evaluated against eligibility criteria. Second, full-text versions of potentially eligible publications were assessed. Two independent reviewers screened at both stages, with disagreements resolved through consensus or adjudication by a third reviewer. Cohen's kappa was calculated to assess the interrater reliability (27). Duplicates were removed prior to screening. Reasons for exclusion at the full-text stage were documented, and the selection process was summarized in a PRISMA flow diagram.

#### Data extraction

Data extraction was performed using a standardized template that captured the study design, setting, baseline patient characteristics, intervention and comparator details, sample size, follow-up duration, and all prespecified efficacy and safety outcomes. Extraction was completed by one reviewer and independently checked by a second reviewer; discrepancies were reconciled by consensus.

#### Risk of bias

Risk of bias for each included RCT was assessed using the Cochrane Risk of Bias 2 (RoB 2) tool (28). Each study was assessed across 5 domains: the randomization process, deviations from intended interventions, missing outcome data, outcome measurement, and selective reporting. Regarding bias, the study was judged as low risk, some concerns, or high risk.

### Outcomes

Relevant outcomes assessed in this analysis included the efficacy outcome, specifically the proportion of patients achieving biochemical control, primarily defined as IGF-I ≤ 1.0 times the upper limit of normal (ULN), with minor variations (one study used <1.3×ULN), and safety outcomes, such as the proportion of patients discontinuing study treatment due to AEs, and the proportion of patients with any TEAE and serious TEAE. Injection-site reactions across studies, including injectable treatments, were summarized descriptively. Treatment discontinuation was mostly reported as discontinuation due to AEs, and safety outcomes were reported as any AEs or any TEAEs, and as serious TEAEs or serious AEs, respectively (Table S1) (29).

### Feasibility assessment

Studies identified in the SLR were assessed regarding the comparability of population, study design, and definition of outcomes. Relevant characteristics of the patient population included in the assessment comprised biochemical control at baseline, medical pretreatment, and failure to respond to standard of care treatment. Networks of evidence were built using all identified studies that reported on the selected outcomes.

### Statistical analysis

#### Input data

Event rates from the treatment arms of each study were used to calculate log odds ratio (OR) and associated standard errors (SE) per study. In the case of zero events, a fixed continuity correction was applied prior to calculating the OR and SE (30).

#### Network meta-analysis

A Bayesian NMA was conducted to estimate the relative effects of pharmacological treatments for acromegaly for each endpoint, estimating effects simultaneously across the entire evidence network. Both fixed- and random-effects models were estimated with adequate convergence. Details on the choice of priors for the fixed- and random effects models are provided in Table S2 (29). The choice of the model serving as the base case was made by evaluating the model fit through the deviance information criterion (DIC), the heterogeneity through the between-study variability *τ*^2^, Higgins *I*^2^ statistics, the within-designs *Q* statistic, and the inconsistency through the between-designs *Q*-statistic. Consistency of direct and indirect evidence was assessed using node-splitting methods (31). Treatment effects were estimated as ORs with corresponding 95% credible intervals (CrIs). These ORs represent the posterior mean of relative effects, with CrIs reflecting uncertainty based on both direct and indirect evidence. Treatment ranking probabilities were summarized using the surface under the cumulative ranking curve (SUCRA) (32), which represents the probability that each treatment ranks among the most effective (or safest) options, with higher values indicating better relative performance across the network. Sensitivity analyses included alternative NMA models and the exclusion of high-risk-of-bias studies or studies leading to high heterogeneity.

#### Software

Analyses were performed using R, Version 4.3.3, (gemtc package) (33) interfaced with JAGS/WinBUGS.

## Results

### Study selection

**Figure 2:**
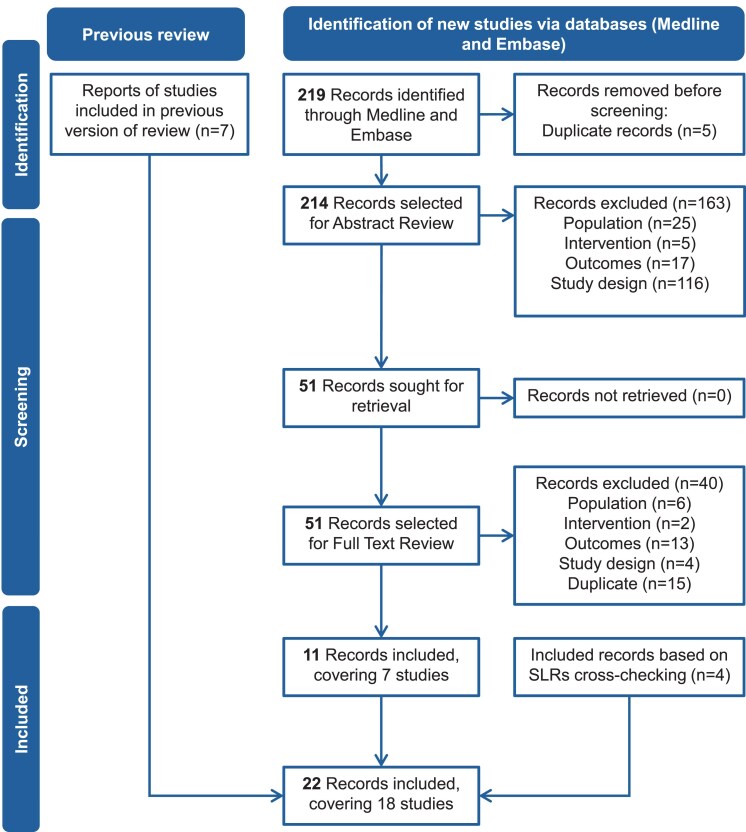
PRISMA flow diagram of study selection for the systematic literature review.

A search of the MEDLINE and Embase databases retrieved 219 records. After removal of duplicates, 214 unique records remained. Following title and abstract screening, 163 records were excluded, and 51 full-text articles were assessed for eligibility. Of these 51, 40 were excluded for reasons such as nonrandomized design, ineligible intervention, or insufficient outcome reporting. From reference checking of other reviews and meta-analyses, 4 records were included (34-37). Seven records from the previous SLR by Leonart et al were also included (9, 38-43). In total, 22 publications reporting 18 unique RCTs met the inclusion criteria and were included in the review. The selection process is summarized in the PRISMA flow diagram (Fig. 2). Cohen's kappa for abstract and full-text selection was 0.72 and 0.78, respectively, thus, moderate interrater reliability was achieved (27).

### Study characteristics

The 18 RCTs included evaluated a range of pharmacological therapies for acromegaly, encompassing cabergoline (in combination with OCT-LAR), LAN-ATG, OCT-LAR, O-OCT octreotide, pasireotide, SC-OCT-D, paltusotine, and pegvisomant. Sample sizes ranged from fewer than 30 participants to more than 150 patients in pivotal phase 3 trials. Follow-up durations varied between 4 weeks and 104 weeks, with most designed to assess short- to medium-term efficacy and safety. Comparator arms included placebo and active controls, depending on trial objectives. Table 2 provides a detailed overview of study characteristics, including interventions, sample size, follow-up duration, and country.

**Table 2:** Summary of studies included in the SLR

Author, year	Study name	Treatment	Dose	Frequency	Route of administration	N	Follow-up (weeks)	Country
Amato 2002 (39)	NR	LAN-PR/SR	30 mg	7-10 days	IM-I	12	104	Italy
		OCT-LAR	10-30 mg	4 wk	I	8		
An 2020 (45)	LANTERN	LAN-ATG	60/90/120 mg	4 wk	SC-I	64	32	China
		LAN-PR	40 mg	7-14 days	IM-I	64		
Andries 2008 (9)	NR	LAN-PR	40 mg	7-14 days	IM-I	64	52	Denmark
		OCT-LAR	10-30 mg	4 wk	IM-I	10		
Biller 2024 (46)	PATHFNDR-2	PAL	20-60 mg	Every day	O	54	22-24	Multinational
		Placebo	NR	NR	NR	57		
Bonert 2020 (44)	NR	SRL-HD + PEG weekly	OCT-LAR: 30 mg	Monthly	I	17	22-32	Unites States
			LAN-LAR: 120 mg	Monthly	I			
			PEG: 40-160 mg	Weekly	I			
		SRL-LD + PEG weekly	OCT-LAR: 10 mg	Monthly	I	23		
			LAN-LAR: 60 mg	Monthly	I			
			PEG: 40-160 mg	Weekly	I			
		SRL-LD + PEG daily	OCT-LAR: 10 mg	Monthly	I	20		
			LAN-LAR: 60 mg	Monthly	I			
			PEG: 15-60 mg	Daily	I			
Colao 2014 (40)	NR	PAS-LAR	40-60 mg	4 wk	I	176	52	27 countries
		OCT-LAR	20-30 mg	4 wk	I	182		
Colao 2019 (47)	NR	OCT-LAR + PEG	OCT-LAR: 40 mg	Every 28 days	IM-I	31	35	Five European countries
			PEG: 70 mg	Weekly	SC-I			
		OCT-LAR + CAB	OCT-LAR: 40 mg	Every 28 days	IM-I	32		
			CAB: 0.25-0.5 mg	Twice weekly - daily	O			
Ferone 2024 (21)	ACROINNOVA 1	OCT sc	20 mg	Monthly	SC-I	47	22/24	Russia, Turkey, Italy, Spain, Germany, United Kingdom, Hungary, Greece, Poland, United States
		Placebo	NR	Monthly	SC-I	24		
		Placebo	NR	Daily	O	28		
Fleseriu 2022 (48), Fleseriu 2021 (49)	MPOWERED	O-OCT	40-80 mg	Daily	O	55	26	Austria, France, Germany, Hungary, Italy, Lithuania, Russia, Serbia, Spain, and the USA
		SRL (OCT-LAR or LAN-ATG)	Same as before trial enrolment	Same as before trial enrolment	I	37		
Gadelha 2024 (20)	PATHFNDR-1	PAL	40-60 mg	Daily	O	30	36	Multinational
		Placebo	NR	Daily	O	28		
Gadelha 2014 (37), Schmid 2016 (34)	PAOLA	PAS	40 mg	Every 28 days	I	65	24	Multinational
		PAS	60 mg	Every 28 days	I	65		
		SRL	OCT-LAR: 30 mg	Continuous treatment	I	68		
			LAN-ATG: 120 mg	Continuous treatment	I			
Ghigo 2009 (41)	NR	PEG	10-40 mg	Every day	I	57	52	13 countries
		OCT-LAR	20-40 mg	4 wk	I	56		
Jenkins 2000 (42)	NR	OCT-LAR	10-30 mg	4 wk	I	18	11-14	United Kingdom
		LAN-PR/SR	30 mg	2 wk	I	11		
Madsen 2011 (35)	NR	iSLR-LD + PEG	OCT-LAR: 6.7-20 mg	4 wk		6	24	Denmark
			LAN-ATG: 24-60 mg	4 wk				
			PEG: 15-30 mg	Twice weekly		12		
Melmed 2010 (43)	NR	LAN-ATG	60-120 mg	4 wk	SC-I	83	4	USA, Europe, Hong Kong
		Placebo	NR	1 injection	I	25		
Neggers 2008 (36)	NR	SRL + PEG	LAN-LAR: NR	Monthly		20	16	The Netherlands
			OCT-LAR: NR	Monthly				
			PEG: 40 mg	Weekly	SC-I			
		SRL + Placebo	LAN-LAR: NR	Monthly		20		
Trainer 2000 (38)	NR	PEG	10 mg	Every day	SC-I	80	12	NR
		PEG	15 mg	Every day	SC-I			
		PEG	20 mg	Every day	SC-I			
		Placebo	NR	Every day	SC-I	32		
		OCT-LAR	40 mg	Every 28 days	IM-I	7		

Abbreviations: ATG, Autogel; CAB, Cabergoline; d, day/days; HD, High dose; I, Injection; IM-I, Intramuscular injection; LAN, Lanreotide; LAN-ATG, Lanreotide Autogel; LAN-LAR, Lanreotide long-acting release; LAN-PR, Lanreotide prolonged release; LAN-SR, Lanreotide sustained release; LAR, Long-acting release; LD, Low dose; mo, month/months; NR, Not reported; O, Oral; OCT-LAR, Octreotide long-acting release; OCT sc, Octreotide subcutaneous; O-OCT, Oral octreotide; PAL, Paltusotine; PAS, Pasireotide; PAS-LAR, Pasireotide long-acting release; PEG, Pegvisomant; SC, Subcutaneous; SC-I, Subcutaneous injection; SLR, Systematic Literature Review; SRL, Injectable somatostatin receptor ligand; wk, week/weeks.

### Risk of bias

Risk of bias was assessed across 5 domains: randomization process, deviations from intended interventions (assigned treatment protocol as designed), missing outcome data, outcome measurement, selective reporting. Risk of bias was assessed for all studies described in a full-text publication (17 studies). Most studies were judged to have some risk of bias, primarily related to the reporting of outcomes or missing data. One study was judged as having a high risk of bias (44), mainly due to potential bias as a result of deviation from the intended intervention (Fig. S1) (29).

### Feasibility assessment

Key population characteristics of the studies identified in the SLR are summarized in Table S3 (29). Transitivity was evaluated via comparable population characteristics (prior treatment, baseline control status); inconsistency via node-splitting (no significant loops, *P* > .05). Three of the 18 studies included only patients who did not have any experience with medical treatment before entering the study (39-41); 4 studies included only medically pretreated patients, but their treatments had to be stopped at least 3 months prior to the start of the study (38, 43, 45, 46); 2 studies included a mix of medically naïve and pretreated patients (9, 42); and 9 studies included patients who were treated with injectable SRLs at baseline (16, 20, 21, 35-37, 44, 47, 48). Six studies included patients who were biochemically controlled at baseline (16, 20, 21, 35, 36, 40, 48, 49); 5 studies included a mix of patients who were biochemically controlled or uncontrolled (9, 42-44, 47); and the remaining studies comprised 6 studies of patients who were biochemically uncontrolled at baseline (37, 38, 41, 45, 46, 49); and 1 study where biochemical control at baseline was not reported (39). Two studies specifically focused on patients who failed to achieve normal IGF-I with injectable SRLs at maximum doses (37, 47). These were considered fundamentally different from the remaining studies that included either medically naïve patients, or patients who were well controlled on the standard of care, or a mix of the 2, but did not require the patients to be unresponsive to injectable SRLs. These studies were therefore excluded from the NMA but are briefly summarized here. The PAOLA study was a multicenter, randomized, phase 3 trial including 198 adults with acromegaly who were biochemically uncontrolled after having received injectable first-generation SRLs (OCT-LAR or LAN-ATG) for ≥6 months. They were randomized to groups as follows: pasireotide 40 mg (*n* = 65), pasireotide 60 mg (*n* = 65), or those who continued previous SRL treatment (*n* = 68). Biochemical control was achieved by 16 (25%) patients receiving pasireotide 40 mg and 17 (26%) patients receiving pasireotide 60 mg at 24 weeks; no patients receiving active control achieved biochemical control (37). The second excluded study was a multicenter, phase 4, open-label study where 63 patients who remained uncontrolled on maximal OCT-LAR dose were randomized to addition of weekly pegvisomant (*n* = 31) or increasing cabergoline doses (*n* = 32). In this study, rates of biochemical control were low in all arms (47).

Although the majority of studies defined biochemical control as serum IGF-I ≤ 1.0×ULN or normalized IGF-I, with or without age- and/or sex-adjustment, one study used a threshold of IGF- I < 1.3×ULN (48). This study was included to maintain comprehensiveness of the evidence base, as the threshold is close to standard definitions and the study met all other inclusion criteria (Table S3) (29).

### Networks

**Figure 3:**
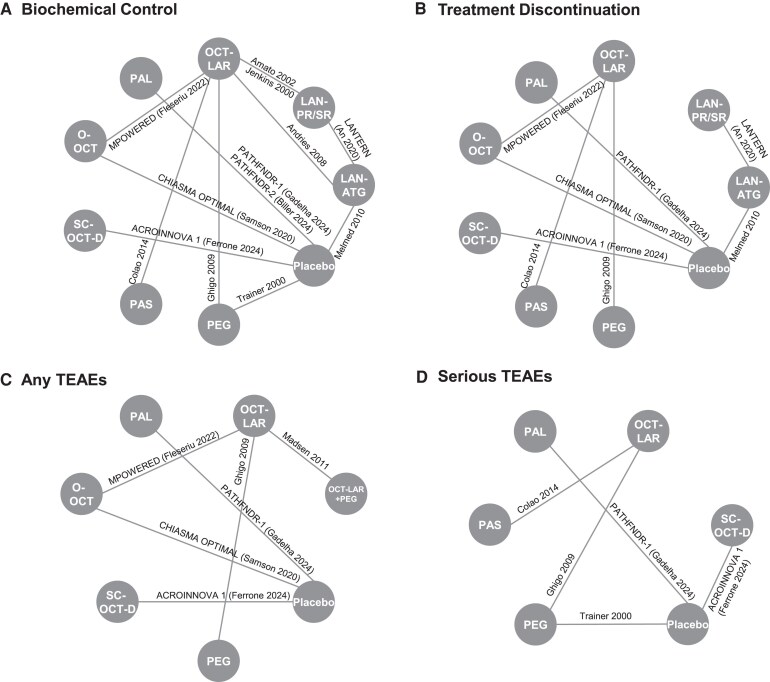
Networks of evidence. (A) Network of evidence for biochemical control, (B) Network of evidence for treatment discontinuation, (C) Network of evidence for any TEAEs, (D) Network of evidence for serious TEAEs. In each network, every node represents one treatment, and edges connect treatments that have been directly compared in at least one trial. Edge labels indicate the trial name or the first author and year of publication. Abbreviations: AE, adverse event; ATG, autogel; LAN, lanreotide; LAR, long-acting release; OCT, octreotide; O-OCT, oral octreotide; PAL, paltusotine; PAS, pasireotide; PEG, pegvisomant; SC-OCT-D, subcutaneous octreotide depot; SR, sustained release; TEAE, treatment-emergent adverse event.

Networks for the 4 endpoints included between 5 and 13 studies (5 studies were excluded from the biochemical control network due to a lack of data (35-37, 44, 47)) and are depicted in Fig. 3. Due to the majority of patients being treated with OCT-LAR during the run-in phase of the MPOWERED study (50), it was assumed that the effect of injectable SRLs in that study was comparable to the effect of OCT-LAR. Similarly, for Madsen 2011 et al, it was assumed that injectable SRL treatment is comparable to OCT-LAR treatment since 83% were treated with OCT-LAR in the injectable SRL-only group (35).

### NMA results

The number of responses/events by treatment arms and study-level ORs with 95% confidence intervals (CIs) served as input data to the NMAs (Table S4) (29). Evaluation of metrics for model-fit, heterogeneity, and inconsistency led to the choice of fixed-effects NMA models for all outcomes (Table S5) (29).

#### Biochemical control

**Figure 4:**
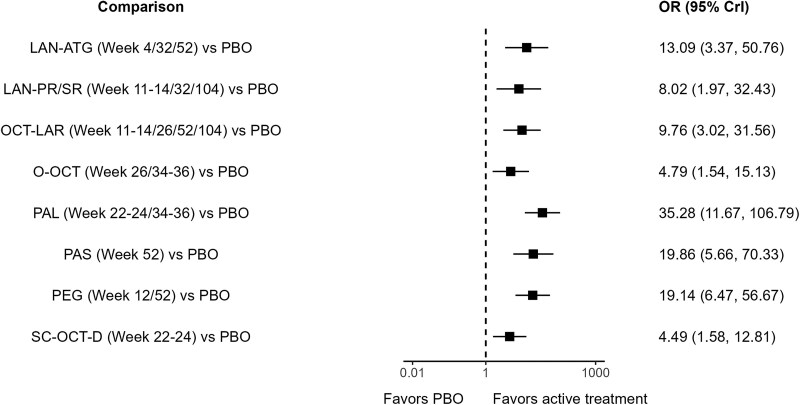
Comparisons vs placebo for biochemical control. Abbreviations: ATG, autogel; CrI, credible interval; LAN, lanreotide; LAR, long-acting release; OCT, octreotide; O-OCT, oral octreotide; OR, odds ratio; PAL, paltusotine; PAS, pasireotide; PBO, placebo; PEG, pegvisomant; PR, prolonged release; SC-OCT-D, subcutaneous octreotide depot; SR, sustained release.

**Figure 5:**
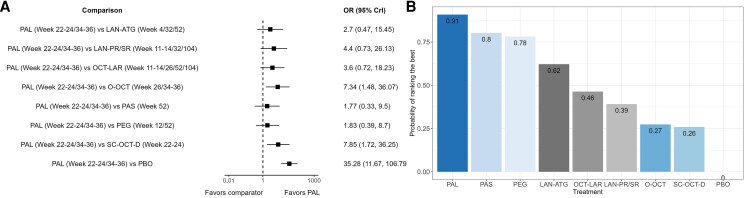
Network meta-analysis result for biochemical control. (A) Forest plot for biochemical control for comparisons with paltusotine. (B) SUCRA plot for the probability of ranking the best for biochemical control. Abbreviations: CrI, credible interval; ATG, autogel; LAN, Lanreotide; LAR, long-acting release; OCT, octreotide; O-OCT, oral octreotide; OR, odds ratio; PAL, paltusotine; PAS, Pasireotide; PBO, placebo; PEG, pegvisomant; PR, prolonged release; SC-OCT-D, subcutaneous octreotide depot; SR, sustained release; SUCRA, surface under the cumulative ranking curve.

Comparisons vs placebo were statistically significant for all treatments with respect to the proportion of patients achieving biochemical control, with the largest effect size with an OR of 35.28 (95% CrI: 11.67-106.79) observed for paltusotine vs placebo (Fig. 4). While the ORs for biochemical control indicated that acromegaly treatments were generally comparable despite varying stabilization/washout periods, results were statistically significant and favored pasireotide vs OCT-LAR (Table S6) (29), and paltusotine vs O-OCT and SC-OCT-D (Table S6, Fig. 5A) (29). The SUCRA plot suggests paltusotine as the treatment with the highest probability of ranking as the most effective to achieve biochemical control (Fig. 5B). In the subgroup NMA of studies in patients who were biochemically controlled on injectable SRLs and switched to a new active monotherapy treatment, results were consistent with the main analysis on biochemical control (Fig. S2A and S2B) (29).

#### Discontinuation due to AEs

**Figure 6:**
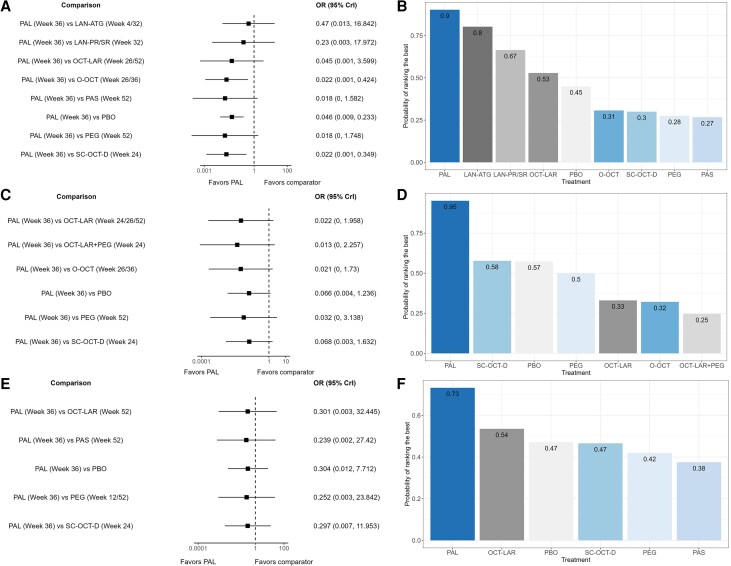
Network meta-analysis results for discontinuation due to AEs, any TEAEs, and serious TEAEs. (A, C, E) Forest plot for the comparisons with paltusotine for discontinuation due to AE, any TEAEs, and serious TEAEs, respectively. (B, D, F) Probability of ranking the best for discontinuation due to AE, any TEAEs, and serious TEAEs, respectively. Abbreviations: ATG, autogel; LAN, lanreotide; LAR, long-acting release; OCT, octreotide; O-OCT, oral octreotide; PAL, paltusotine; PAS, Pasireotide; PBO, placebo; PEG, pegvisomant; PR, prolonged release; SC-OCT-D, subcutaneous octreotide depot; SR, sustained release.

Acromegaly treatments were comparable regarding discontinuation due to AEs, except for paltusotine, which showed a statistically significant lower discontinuation rate due to AEs vs O-OCT, SC-OCT-D and placebo, with an OR (95% CrI) of 0.02 (0.00-0.42), 0.02 (0.00-0.34) and 0.05 (0.01-0.23), respectively (Table S7, Fig. 6A) (29). The SUCRA values indicated that paltusotine ranked the best for discontinuation due to AEs (Fig. 6B). In the subgroup NMA of studies in patients with biochemical control on injectable SRLs and who switched to a new active monotherapy treatment, results were consistent with the main analysis (Fig. S2C and S2D) (29).

#### Safety outcomes

Treatments for acromegaly were comparable regarding any TEAEs (Table S8) (29) and serious TEAEs (Table S9) (29). The corresponding forest plot of paltusotine vs all treatments is shown in Fig. 6C for any TEAEs and Fig. 6E for serious TEAEs. SUCRA values indicated that paltusotine ranked the best for safety outcomes, however, credible intervals of the relative effects largely overlapped across treatments (Fig. 6D and 6F, Tables S8 and S9) (29). In the subgroup NMA of studies in patients who were biochemically controlled on injectable SRLs and who switched to a new active monotherapy treatment, results were consistent with the main analysis on any TEAEs and serious TEAEs (Fig. S2E-S2H) (29). Injection-site reactions were reported only for injectable agents, ranging from 2% to 9% of patients in some studies to 63% in others (Table S10) (29).

## Discussion

This systematic literature review and network meta-analysis highlights the evolving landscape in medical therapy for acromegaly, offering a comprehensive synthesis of randomized controlled trial evidence for pharmacological therapies in adults with acromegaly who were either medically naïve, pretreated and washed out, or controlled on the standard of care. The analysis incorporates evidence on the efficacy and safety of both established injectable SRLs, pegvisomant, and recently reported pivotal trials of oral paltusotine, O-OCT, and SC-OCT-D that were not included in prior SLRs and (network) meta-analyses (22-24, 51). By integrating direct and indirect evidence, the NMA allows a robust comparison of relative efficacy and safety across all included agents. The NMA revealed similar efficacy and safety outcomes for most treatments. SUCRA graphs suggested that the likelihood of ranking best for efficacy and safety was consistently higher for paltusotine. Differences in ORs reflect relative effects within the evidence network and should be interpreted in the context of trial size, network structure, and overall uncertainty. Specifically, paltusotine showed statistically superior biochemical control vs O-OCT (OR 7.34, 95% CrI 1.48-36.07) and SC-OCT-D (OR 7.85, 95% CrI 1.72-36.25), plus lower AE discontinuations (OR for the comparison vs O-OCT 0.022, 95% CrI 0.001-0.424; OR for the comparison vs SC-OCT-D 0.022, 95% CrI 0.001-0.349), and pasireotide was superior to OCT-LAR for biochemical control (OR 2.03, 95% CrI 1.29-3.23). Although some studies required pretreatment response to short-acting octreotide/lanreotide or included >50% of patients with prior irradiation, potentially inducing a bias toward older therapies, paltusotine consistently ranked highest across endpoints, demonstrating robust performance despite these prespecified enrollment factors.

A previous NMA of RCTs by Leonart et al showed favorability of pegvisomant and pasireotide vs standard injectable SRLs for biochemical control, although CrIs were wide (22). Another indirect treatment comparison analysis by Qiao et al which included RCTs and observational studies and employed simulated trials, found that pegvisomant and pasireotide exhibited statistically significant superior biochemical control compared with standard injectable SRLs in subgroups with partial biochemical control at baseline (24). The most recent NMA also showed that pasireotide LAR and pegvisomant were the most effective treatments for IGF-I normalization (23). As mentioned, the analysis was completed before the latest clinical trials with SC-OCT-D and oral paltusotine were published, creating the need for an updated NMA such as the one presented herein. Although differences in the approaches and studies preclude a quantitative comparison of the results, the findings of previous NMAs are in line with results presented herein where pegvisomant and pasireotide achieve a higher probability of ranking the best compared with LAN-ATG, OCT-LAR, and LAN PR/SR. Importantly, the recent Kaparounaki et al (23) NMA included both randomized and observational studies, but did not include paltusotine or SC-OCT-D, and safety outcomes were reported descriptively without a formal comparison. In contrast, our study included only RCTs, providing a controlled and methodologically rigorous assessment, while also integrating emerging agents to offer a comprehensive up-to-date evaluation of the acromegaly treatment landscape. Overall, the findings of Leonart et al (22), Qiao et al (24), and Kaparounaki et al (23) are directionally consistent with our results, reinforcing the strong efficacy of pegvisomant and pasireotide, while our analysis extends the comparative evidence to include the oral agent paltusotine, which demonstrates comparable efficacy and tolerability to injectable SRLs, pegvisomant, and pasireotide. The heterogeneity observed in the placebo control arms likely has influenced the comparative efficacy estimates between active treatments, beyond what is captured by the absolute response rates. Variations in study design, patient populations, baseline disease severity, and outcome definitions likely contributed to these differences.

For years, injectable SRLs with a high affinity for SST2, including OCT-LAR and LAN-ATG, have been the cornerstone of medical therapy (52). Pasireotide, an SRL with broader receptor affinity (notably SST5), has demonstrated improved biochemical efficacy compared with OCT-LAR in head-to-head trials and NMAs (24, 40). However, pasireotide is associated with a high incidence of hyperglycemia and new-onset diabetes, which may limit its use, especially in patients with pre-existing glucose intolerance (40). Pegvisomant effectively normalizes IGF-I levels, as both a monotherapy and as part of a combination regimen. Unlike SRLs, pegvisomant does not reduce tumor size or suppress GH secretion, necessitating regular magnetic resonance imaging surveillance for tumor progression (53). Pegvisomant is particularly valuable in patients who are unresponsive to SRLs or with contraindications to SRLs, and in those with comorbid diabetes (54). Liver function monitoring is required due to rare but potentially significant transaminase elevations (55). Twice a day octreotide capsules have recently offered an alternative to injectable formulations for the continuing maintenance of patients who have already achieved biochemical control with injectable SRLs (15); however, twice a day dosing may pose challenges in terms of adherence and convenience. SC-OCT-D is a subcutaneous octreotide depot formulation (56) designed to improve bioavailability and patient convenience compared with standard long-acting injectable SRLs (19).

While AEs observed with SRLs are generally mild and include gastrointestinal symptoms and cholelithiasis, cholecystitis remains rare (57). However, multiple studies have demonstrated a persistent symptom burden among patients with acromegaly who are treated with long-acting SRLs. Patients frequently experience symptoms such as fatigue, joint pain, and swelling (58), and worsening of symptoms toward the end of the injection cycle, suggesting that long-acting SRLs may not provide consistent symptom control throughout the dosing interval (59). In a large survey of 195 patients receiving long-acting SRLs (octreotide or lanreotide), over 70% reported ongoing acromegaly symptoms despite treatment (60). Notably, 52% experienced symptom worsening toward the end of the dosing interval, which significantly impacted daily functioning and emotional well-being (60). A recent prospective study indicates that patients experience symptoms of acromegaly toward the end of the SRL injection cycle, which is correlated with an increase in IGF-I levels related to the time since SRL injection (61).

Injection-site reactions, including pain, nodules, and swelling, are frequent and burdensome, and patients have expressed a preference for noninjectable treatment options (49). Although we have included a table to compare the injection-site reactions reported with injectable therapies, a formal NMA could not be conducted for this outcome, as injection-site reactions are not applicable to oral treatments. The above-mentioned issues with long-acting injectable medications highlight a meaningful advantage for oral therapies. Indeed, patients have consistently expressed a preference for noninjectable options and a desire for improved symptom control, underscoring the potential value of emerging oral therapies in addressing unmet needs in tolerability and convenience (49).

Paltusotine, an oral SRL not included in previous NMAs, is administered once daily and has demonstrated robust biochemical control in patients switched from depot SRLs as well as in those who are untreated or inadequately controlled (20). Biochemical control was reached in 83.3% of the patients who were treated with paltusotine in a phase 3 RCT (PATHFNDR-1), compared with 3.6% of patients on placebo (20). In PATHFNDR-2, a placebo-controlled phase 3 trial in medically naïve or previously treated patients with biochemically uncontrolled acromegaly at baseline, 55.6% of the patients treated with paltusotine achieved biochemical control vs 5.3% for placebo, supporting the efficacy and safety of paltusotine in a broader patient population (46). According to the NMA, biochemical control with paltusotine was comparable to that of pasireotide, pegvisomant, LAN-ATG, and OCT-LAR and significantly superior to O-OCT and SC-OCT-D, and statistically lower rates of treatment discontinuation due to AEs were observed with paltusotine compared with O-OCT and SC-OCT-D. These results support the use of paltusotine as an effective and well-tolerated noninjectable alternative with the potential to improve patient adherence and convenience.

### Limitations

Several factors should be considered when interpreting these results. Heterogeneity among the patient populations in the trials included in the NMA leads to bias in indirect treatment comparisons, although the Bayesian NMA framework partially accounts for these. Prior SRL responder populations dominate some trials and may bias effect estimates toward injectable SRLs and oral SRLs similarly. Most of the studies included at least a subset of patients who had previously responded to an SST2 agonist, possibly enhancing the relative efficacy of this class of drugs compared to the broader-acting SRL pasireotide or a GH receptor antagonist. The rate of IGF-I control among the placebo groups differed widely among studies, likely influencing the findings. Outcome definitions allowed for some variation, such as biochemical control being defined as IGF-I ≤ 1.0×ULN (consensus guideline target) or IGF-I < 1.3×ULN, and discontinuation due to AEs or TEAEs leading to study treatment withdrawal, but were considered sufficiently harmonized. Follow-up durations varied across the trials included in the NMA, which could introduce potential bias into the indirect treatment comparisons. However, since each trial was designed to evaluate efficacy at its respective endpoint, efficacy outcomes are expected to reflect treatment effects regardless of differences in study length. In addition, the studies included herein span different time periods, during which changes in clinical practice, patient management, and assay methods may have influenced outcomes, potentially introducing bias in the indirect comparisons. Although SUCRA scores simplify ranking information, they can carry uncertainty, particularly when treatment effects are similar, and they may be sensitive to heterogeneity, sparse data, and model assumptions, especially in networks relying heavily on indirect comparisons. Therefore, they should be interpreted in the context of uncertainty in the underlying treatment effects. Finally, pituitary tumor control is an important clinical outcome in acromegaly; however, it was not assessed in our analysis because most RCTs included herein were of short duration and did not systematically evaluate changes in tumor size.

The finding of heterogeneity in design and methods and the surprising differences in placebo arms even among RCTs specifically selected to meet key criteria and excluding bias, is instructive. Standardization of design and methods in RCTs on emerging therapies for acromegaly is warranted for optimal comparative analysis of the therapeutic options. A uniform approach to the construction of RCTs on therapies for rare disease may enhance their collective impact.

## Conclusion

This updated evidence synthesis confirms the efficacy and safety of established injectable SRLs and emerging oral therapies for acromegaly. Among the drugs not compared in previously published literature, paltusotine offers a promising oral alternative with efficacy comparable to that of injectable SRLs and a favorable tolerability profile, potentially improving patient convenience and adherence. This NMA contextualizes new medical treatments such as paltusotine and SC-OCT-D that were not included in previous NMAs within the broader treatment landscape for patients with acromegaly who are either medically naïve, pretreated and washed out, or controlled on the standard of care. These findings may inform clinical decision-making and guideline development, supporting personalized treatment selection based on efficacy, safety, and patient preference. Future studies, including long-term extension trials and real-world observational data, will be valuable to validate the findings of this NMA in clinical practice and to fully characterize the comparative effectiveness, durability, and safety of paltusotine and other therapies for acromegaly over extended treatment periods.

## Data Availability

Datasets generated and analyzed during the current study are not publicly available but are available from the corresponding author upon reasonable request.
